# My Obstetric Experience in Twenty-nine Years Practice of Medicine with Some Suggestions

**Published:** 1914-04

**Authors:** R. G. Loyd

**Affiliations:** Royse City, Texas


For Texas Medical Journal.

### My Obstetric Experience in Twenty-nine Years Practice of Medicine With Some Suggestions.

BY R. G. LOYD, M. D., ROYSE CITY, TEXAS.

Inasmuch as I have not kept a record of all my cases, I shall have to speak largely from memory. I dare say that every young man starting into the field of general practice, will not soon forget the experience he had with his first cases of labor, even though his mind may not be so clear in what transpires in later years.

I began the practice of medicine in the county in which I now live in 1884, and with the exception of eight months, five of which I was in school, I have continued to live, following the same calling up to the present time. I will say in the first place that my obstetrical practice has never been an overwhelming one; however, I presume it has been about on an average with the small town and rural physicians. I never used instruments nor had them used in any case of labor of my own; that is, where I was first called and had charge of the case; however, I have assisted in several instrumental deliveries. I have lost two mothers in my entire experience in labor cases. The first was Mrs. E., in 1885, the second year I practiced medicine. I was called to see her about one week before the period of her confinement. She had a well developed case of lobar pneumonia; she was delivered of a living child, but died three days later from the attack of pneumonia. The other case was the wife of a wood chopper who had previously resided for some time in East Fork bottom. I treated her at intervals for chronic malaria for two or three months prior to her confinement. She also gave birth at term to a living child, but died three weeks later of malaria and perhaps other infection. With the two exceptions given I have never had a fatal case in labor, or during the puerperal stage; however, I have had quite a number of still births. In giving my experience and record in that line of practice to the medical profession, I do so without any spirit of boasting; nor do I attribute it to any special skill on my part; nevertheless, it is a record of which I feel proud.

*Now to the Suggestions.* The point I wish to make and emphasize is to give nature a fair chance. The fact that reproduction is a natural law, and the further fact that normal labor is a natural phenomenon in the female sex, we should not unnecessarily interfere with that function.

When I am called to a case and after preliminary examination I find the woman to be in labor, the first thing I do is to try to get the full confidence of my patient. A great many of our women at this day and time are of a neurasthenic type, easily disturbed and discouraged, especially if it a young woman in her first labor, and the labor is apt to be more or less protracted. Now at this point I want to say that according to my experience in these cases it sometimes occurs that nature will finally cease to act or respond to a great extent; we will have uterine inertia by the patient retracting the muscles and resisting the labor paine due to a dread of pain and misery caused by the uterine contractions. At this stage is where a woman needs encouragement and help most. Some physicians, especially young men with limited experience, are too much prone, I fear, to the use of forceps in order to give them a reputation, to curtail time or for other reasons. According to my observation instrumental deliveries are fraught with considerable danger, to both mother and child, even though they could always be used by skillful hands. During my professional life I have had many cases where both patient and members of the family became very much disturbed and discouraged, so to speak, would begin to doubt the ability of the woman to give birth to the child. If I had shown any indications that way myself, and only intimated that the case was not progressing as favorably or as rapidly as it should, and that it might become necessary to use instruments, business would likely go to picking up in that direction very soon, and more than likely annul to a great extent any further voluntary effort on the part of the patient. In such cases I boost and encourage them in every way I can, telling them it is a natural case of labor, that it was intended by nature for some cases to be slow in order to prepare the soft parts for the strain to which they have been subjected.

I never had but two injured perineums which required suturing, and, strange to say, they both occurred within a few weeks of each other; one was complete, the other was only partial. Now if the experience I have given and what little I have said in somewhat of a disconnected way, should prove of any benefit or help to anyone in the medical profession, I shall feel amply repaid for the time spent in writing it. And should it not go to the waste-basket via the editor's office, I may in the near future give an article on the technique of my labor cases, including premature labors, also the management of mother and child in parturition and during the puerperal stage.

